# Functional and Mechanistic Insights of 3-Hydroxybutyrate (3-OBA) in Bladder Cancer

**DOI:** 10.3390/molecules30234624

**Published:** 2025-12-02

**Authors:** Ana Silva, Ana Mafalda Félix, Céline S. Gonçalves, Adhemar Longatto-Filho, Fátima Baltazar, Julieta Afonso

**Affiliations:** 1 Life and Health Sciences Research Institute (ICVS), University of Minho, Campus of Gualtar, 4710-057 Braga, Portugalfbaltazar@med.uminho.pt (F.B.); 2 ICVS/3B’s—PT Government Associate Laboratory, 4710-057 Braga, Portugal; 3 Laboratory of Medical Investigation (LIM14), Faculty of Medicine, São Paulo State University, São Paulo 01049-010, SP, Brazil; 4 Molecular Oncology Research Center, Barretos Cancer Hospital, Barretos 14784-400, SP, Brazil

**Keywords:** bladder cancer, 3-hydroxybutyrate, lactate, GPR81, monocarboxylate transporters

## Abstract

Bladder cancer (BC), particularly muscle-invasive urothelial bladder carcinoma (UBC), remains a clinical challenge due to frequent recurrence, chemoresistance, and limited treatment options. This study investigates the functional and mechanistic insights of 3-hydroxybutyrate (3-OBA), a ketone body with known metabolic and epigenetic roles, in muscle-invasive UBC models. 3-OBA significantly inhibited cell viability, proliferation, migration, and invasion in T24 and HT1376 cell lines in a dose-dependent manner. In vivo, 3-OBA impaired tumor growth and angiogenesis in the chick chorioallantoic membrane model. Mechanistically, 3-OBA did not alter the expression of the G-protein-coupled lactate receptor GPR81 or associated markers (phospho-ERK1/2, LDHA, MCT1/4, CD147), indicating its antitumor effects are GPR81-independent. Moreover, extracellular lactate modulation upon 3-OBA treatment varied between cell lines, with HT1376 cells showing reduced lactate production under nutrient deprivation, suggesting cell-specific metabolic responses to 3-OBA. These findings highlight 3-OBA’s potential as a metabolic modulator with antitumor efficacy in UBC, particularly in metabolically constrained tumors. However, its dual role—as both a potential energy source and therapeutic agent—demands context-specific investigation. Future studies should focus on patient stratification and preclinical validation to clarify 3-OBA’s therapeutic window and mechanism of action in bladder cancer.

## 1. Introduction

Bladder cancer (BC) ranks as the second most common malignancy of the genitourinary system, surpassed only by prostate cancer. Global statistics from 2022 reported BC as the ninth and the thirteenth leading cause of cancer-related diagnosis and mortality, respectively (613,279 new cases and 220,349 deaths). Risk factors for BC typically include smoking and occupational exposure to aromatic amines, with the disease being more prevalent among older men [1,2]. Urothelial bladder carcinoma (UBC) accounts for about 90% of all BC cases [3]. The recurrent and progressive nature of non-muscle invasive (NMI) tumors (about 3/4 of all UBC), coupled with the intrinsic or acquired resistance to cisplatin-based chemotherapy in muscle-invasive cases (about 1/4 of all UBC), makes UBC care the most expensive among all malignancies [4]. Although immunotherapy [5] and other targeted treatments [6] offer promising avenues, their efficacy remains hindered by frequent occurrences of therapeutic resistance [7,8]. NMI-UBC patients generally display a relatively favorable five-year survival rate, but this dramatically declines to 50% when muscle invasion is present, and to 10% in cases of metastatic progression [9]. Hence, novel anticancer agents with superior efficacy and improved selectivity compared to existing treatments are crucial for advancing patient care and enhancing survival outcomes.

3-Hydroxybutyrate (3-OBA) or beta-hydroxybutyrate is a hydroxy acid with the molecular formula C4H8O3, existing in two enantiomeric forms, D-3-hydroxybutyrate and L-3-hydroxybutyrate; the D-form is biologically active in humans [10]. Conditions of low carbohydrate availability cause endogenous production of this ketone body in the mitochondria of hepatocytes, from free fatty acids oxidation during fatty acid metabolism. It then travels through the bloodstream, reaching extrahepatic tissues to be transformed into acetyl-Coenzyme A, entering the Krebs cycle for further metabolism. It serves as an alternate energy source for the brain, heart, and skeletal muscles when glucose levels are insufficient [11]. The baseline concentration of 3-OBA in human serum typically resides in the low micromolar range, but its levels significantly increase under specific metabolic conditions like fasting (from several hundred micromoles after 12–16 h of fasting to 6–8 mM after extended starvation), intense exercise (1–2 mM after 90 min of strenuous physical activity) or ketogenic diet (above 2 mM). These fluctuations demonstrate 3-OBA’s critical role as a metabolic fuel during periods of reduced carbohydrate availability or increased energy demands. Moreover, modest rises in 3-OBA levels modulate signaling pathways involved in cell growth, proliferation and antioxidant defense [11,12,13].

3-OBA has gained significant interest in various fields: it can serve as a precursor in the synthesis of biodegradable polymers [14], it can be used as a biomarker for ketosis [15] and has many other medical implications and applications in conditions like diabetes [16], epilepsy [17] and Alzheimer’s disease [18]. In cancer, it plays mainly epigenetic and metabolic roles, although also encompassing other signaling pathways [10,12]. 3HB is a natural inhibitor of class I and II a histone deacetylases (HDAC), which can activate tumor-suppressor genes and suppress tumor-promoting pathways [19], but it can also enhance the expression of pro-metastatic genes in specific contexts [20]. On the other hand, cancer cells rely mostly on glucose to obtain energy (Warburg effect), and 3-OBA-based interventions, such as ketogenic diets or exogenous ketones, may starve glucose-dependent tumors while nourishing healthy tissues. Yet, due to the metabolic flexibility of cancer cells, ketone bodies may provide substrates for biosynthesis in nutrient-deprived environments, aiding cancer survival [21,22]. Thus, 3-OBA has a dual role in cancer biology, with potential to both suppress and support cancer growth depending on the context, making it a promising but nuanced area for therapeutic exploitation.

Some authors have used 3-OBA as an antagonist of the G-protein coupled receptor (GPCR) 81, which has lactate as an endogenous ligand [23,24,25]. This lactate-sensing GPCR is composed of seven transmembrane domains that trigger intracellular signaling cascades upon lactate binding mainly by inhibiting adenylate cyclase activity, leading to decreased cyclic AMP (cAMP) levels within the cell. The reduction in cAMP impacts various downstream signaling pathways, including metabolic regulation, inflammation, and cell survival, particularly in the context of cancer progression and tumor microenvironment adaptation [26]. Thus, GPR81 inhibition may constitute a factual anticancer approach, since lactate is the main metabolite exported, through monocarboxylate transporters (MCTs), from glycolytic cancer cells, supporting both microenvironmental acidosis and GPR81-mediated signaling [27]. GPR81 expression has been shown to be upregulated in tumor tissues and cell lines [28], especially in breast cancer [29,30]. However, GPR81 role in cancer remains elusive, including in bladder cancer, and current experimental evidence cannot conclusively support 3-OBA antagonistic activity [31].

The aim of this study was to pre-clinically explore the functional effects of 3-OBA treatment in muscle invasive UBC cell lines, and to shed light on the underlying mechanisms of such treatment, namely its influence in GPR81-mediated signaling. 3-OBA has shown promising anticancer activity both in UBC cell lines and UBC cell lines-derived tumors, which seemed to be dose- and time-dependent, and occur through a GPR81-nondependent mechanism. Thus, 3-OBA did not act as a GPR81 antagonist in the setting of UBC.

## 2. Results

### 2.1. Effects of 3-OBA Treatment in Muscle-Invasive UBC Cell Lines

**Figure 1:**
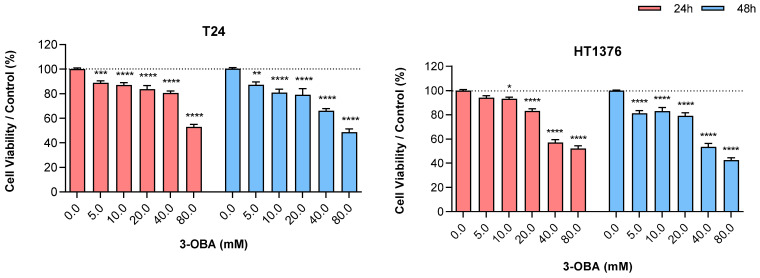
Effect of increasing concentrations of 3-OBA treatment for 24 and 48 h on the viability of T24 and HT1376 cell lines. Results are presented as normalized means ± standard error of the mean (SEM) of at least three independent assays. Statistical significance was estimated by two-way ANOVA followed by Tukey’s multiple comparison post hoc test. * *p* < 0.05, ** *p* < 0.01, *** *p* < 0.005 and **** *p* < 0.001 for each 3-OBA concentration versus control condition (0.0 mM 3-OBA).

T24 and HT1376 muscle-invasive UBC cell lines were treated with increasing concentrations of 3-OBA, ranging from 0.0 to 80.0 mM, during 24 h and 48 h. There was a significant decrease in cell viability upon 3-OBA treatment in a dose-dependent manner. The exposure time did not influence 3-OBA impact (Figure 1). We chose to use 5 mM and 10 mM concentrations in the remaining assays, as these were the initial doses that promoted a significant inhibitory effect in the viability of both cell lines.

**Figure 2:**
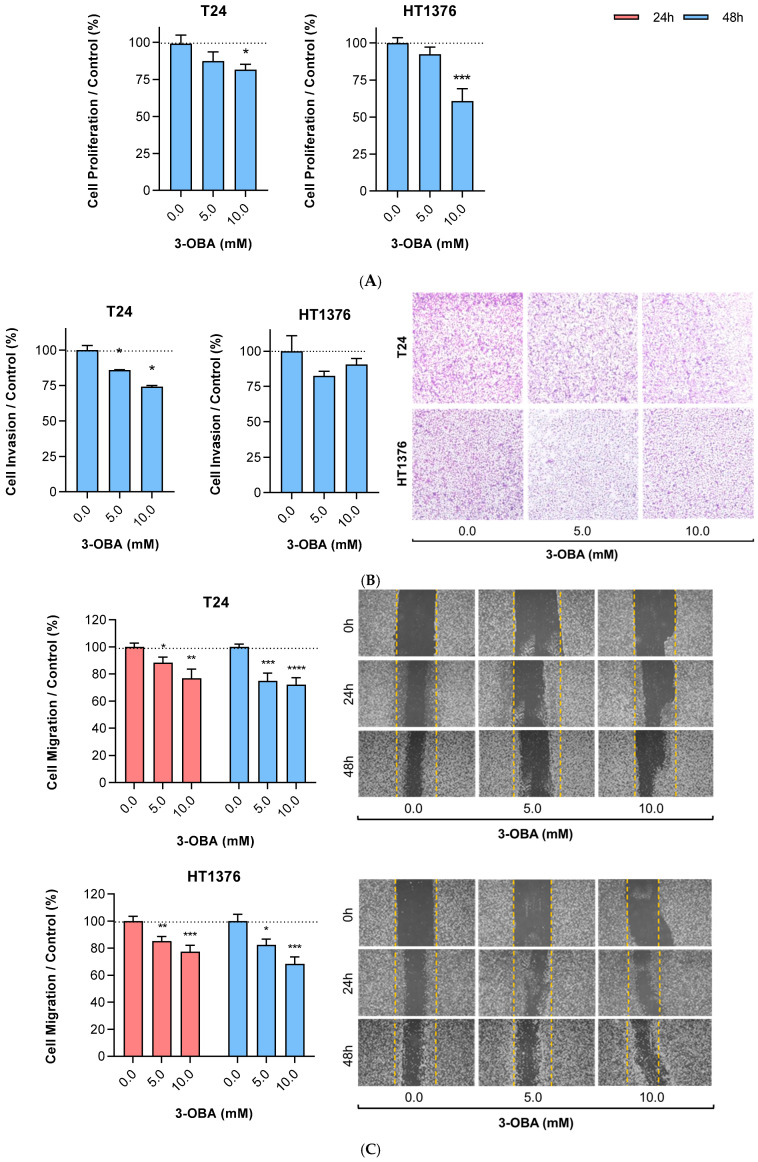
Effect of increasing concentrations of 3-OBA treatment on the proliferation (**A**), invasion (**B**) and migration (**C**) of T24 and HT1376 cell lines for 24 (**C**) and 48 h (**A**–**C**). Representative images of each conditions for the invasion (**B**) and migration (**C**) assays are shown. Results are presented as normalized means ± standard error of the mean (SEM) of at least three independent assays. Statistical significance was estimated by one- or two-way ANOVA followed by Dunnett’s or Tukey’s multiple comparison post hoc tests, respectively. * *p* < 0.05, ** *p* < 0.01, *** *p* < 0.005 and **** *p* < 0.001 for each 3-OBA concentration versus control condition (0.0 mM 3-OBA).

3-OBA treatment for 48 h decreased the proliferative ability of the cells; the differences were significant when a 10 mM dose was administered (Figure 2A). Although we observed a similar inhibitory pattern in the invasion assay, T24 cells appeared to be more sensitive to the drug, showing significant differences when compared to the control condition for the two tested concentrations (Figure 2B). In the same line, a significant decrease in T24 and HT1376 cells’ migration was noted after both 24 h and 48 h of 3-OBA treatment, although no differences were observed regarding the different exposure lengths (Figure 2C), which corroborates time-independence already observed in the cell viability assay (Figure 1).

**Figure 3:**
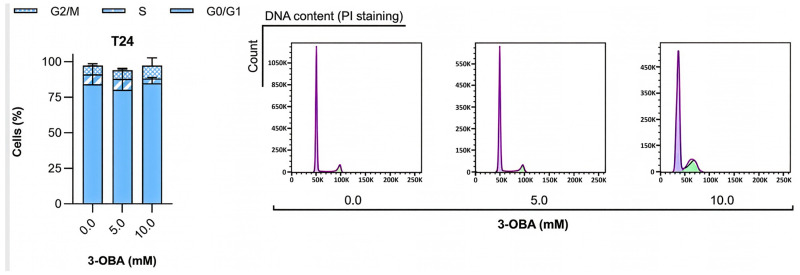

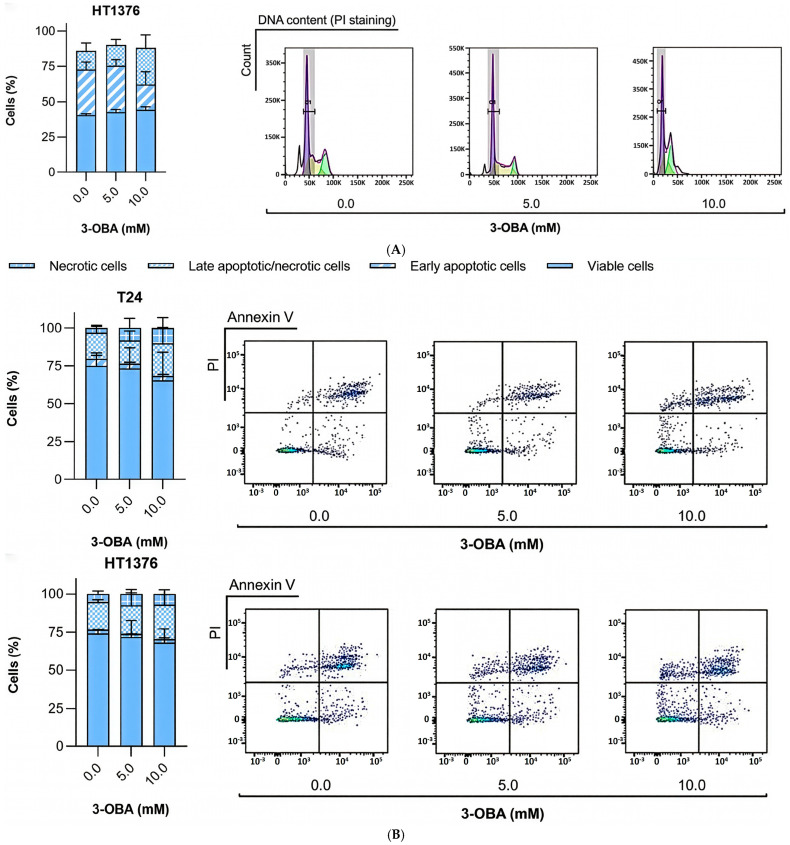
Effect of increasing concentrations of 3-OBA treatment for 48 h on the cell cycle distribution (**A**) and on cell death (**B**) of T24 and HT1376 cell lines. Representative cell cycle profiles and dot plots for the cell cycle (**A**) and the cell death (**B**) assays, respectively, are shown. Results are presented as normalized means ± SEM of at least three independent assays. Statistical significance was estimated by two-way ANOVA followed by Bonferroni multiple comparison post hoc test. *p* > 0.05 for each 3-OBA concentration versus control condition (0.0 mM 3-OBA).

Analysis of cell cycle distribution upon 3-OBA treatment for 48 h revealed a tendency (although not significant) for UCB cells’ accumulation in G0/G1 and G2/M phases, with a concurrent decrease in cells in the S phase, mainly when the cells were treated with 10 mM 3-OBA (Figure 3A). Regarding analysis of cell death occurrence, 3-OBA treatment decreased the viable cells’ fraction (although not significantly) in a concentration-dependent manner, concomitant with an increase in the percentage of late apoptotic/necrotic cells (Figure 3B).

### 2.2. Effects of 3-OBA Treatment in Muscle-Invasive UBC Cell Lines-Derived Tumors

**Figure 4:**
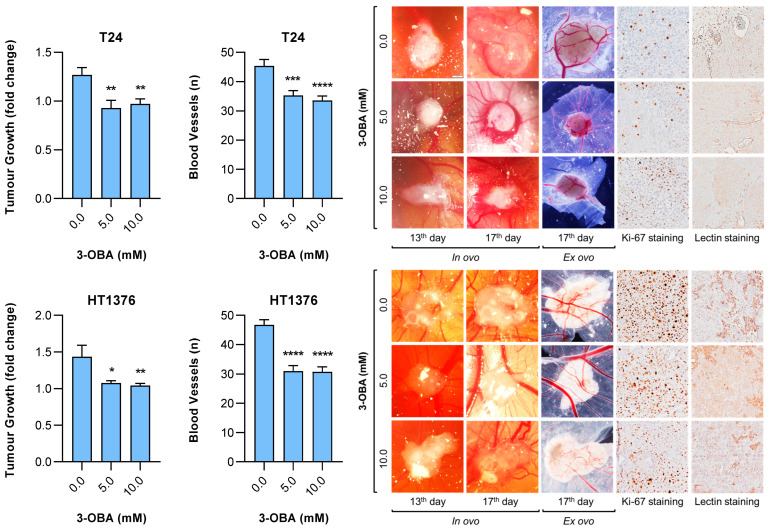
Effect of increasing concentrations of 3-OBA treatment for 96 h hours on tumor growth and blood vessels formation of T24 and HT1376 cell lines-derived tumors. Representative images of in ovo and ex ovo tumors on the chick chorioallantoic membrane at the beginning (13th day post egg incubation) and end (17th day post egg incubation) of 3-OBA treatment are shown, together with Ki-67 (stains proliferative cells) and lectin (stains blood microvessels) immunostaining of excised tumor-derived tissue sections (original magnification 100×). Results are presented as normalized means ± SEM regarding the following number of tumors analyzed per each condition: T24, 0.0 mM 3-OBA—17 tumors, 5.0 and 10 mM 3-OBA—20 tumors; HT1376, 0.0 and 10.0 mM 3-OBA—17 tumors, 5.0 mM 3-OBA—15 tumors. Statistical significance was estimated by one-way ANOVA followed by Dunnets’s multiple comparison post hoc test. * *p* < 0.05, ** *p* < 0.01, *** *p* < 0.005 and **** *p* < 0.001 for each 3-OBA concentration versus control condition (0.0 mM 3-OBA).

We used the chick chorioallantoic membrane (CAM) assay to test the consequences of 3-OBA treatment in a suitable in vivo model. T24 and HT1376 cells were injected at the surface of the CAM on day nine post-egg incubation, and after confirming tumor formation/implantation at day thirteen, the treatment was administered to the different control and test groups. A significant decrease in tumor growth and blood vessels formation was observed on day seventeen in both test groups (5 and 10 mM 3-OBA treatment), when compared to the control condition for the two cell lines-derived tumors. In accordance, we observed a qualitative decrease in cell proliferation and neovascularization, as noted by the immunostaining of Ki-67 and lectin in excised tumor-derived tissue sections. No differences were observed among the two test groups (Figure 4).

### 2.3. Mechanistic Insights of 3-OBA Treatment in Muscle-Invasive UBC Cell Lines

**Figure 5:**
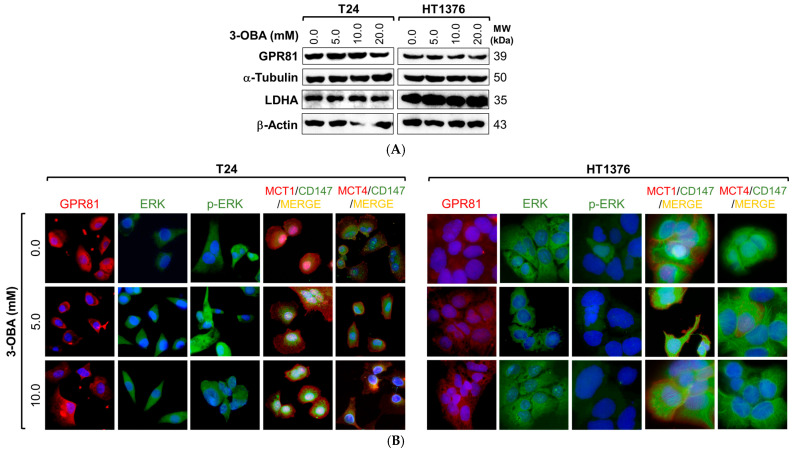
Effect of increasing concentrations of 3-OBA treatment for 48 h on the expression levels of GPR81 (**A**,**B**), lactate dehydrogenase A (**A**), total ERK1/2 (**B**), phospho-ERK1/2 (**B**), MCT1 and 4 and their chaperone CD147 (**B**), in T24 and HT1376 cell lines. The Western blot results (**A**) are representative of similar immunoblots from three independent cell lysates (quantification of the results shown in Figure S1). In (**B**), blue color results from counterstaining of the cell nuclei with DAPI; immunofluorescence images were obtained at 400× amplification (scale bar 50 μm).

In order to investigate a putative link between 3-OBA effects and the lactate receptor GPR81, we started by analyzing the expression of GPR81 and related proteins upon 3-OBA treatment (we also analyzed the clinicopathological and prognostic significance of GPR81 expression in tissue samples from UBC patients—in Supplementary Materials, Figure S2 and Table S1). Immunoblots (Figure 5A) did not reveal any significant difference (quantification of the results shown in Figure S1) between the control and treatment conditions regarding GPR81 and lactate dehydrogenase A (responsible for pyruvate/lactate inter-conversion); immunofluorescence images (Figure 5B) for GPR81 corroborate the Western blot results. We also performed immunofluorescence for other GPR81-related proteins, namely total ERK 1/2 and phospho-ERK1/ERK2 (extracellular signal-regulated kinase, modulated by GPR81), MCT1 and 4 (lactate and 3-OBA transmembrane transporters) and their obligatory chaperone CD147 (Figure 5B). Similarly, no qualitative differences were found among the different treatment conditions.

**Figure 6:**
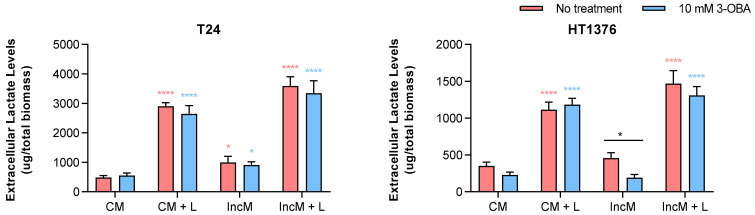
Effect of 10 mM 3-OBA treatment for 48 h on extracellular lactate levels of T24 and HT1376 cell lines, when incubated in culture medium with or without glucose, glutamine, and pyruvate (complete—CM or incomplete—IncM—culture medium, respectively), with (CM + L) or without (IncM + L) lactate. Results are presented as normalized means ± SEM of at least three independent assays. Statistical significance was estimated by two-way ANOVA followed by Tukey’s multiple comparison post hoc test, or by the unpaired two-tailed Student’s *t*-test. * *p* < 0.05 and **** *p* < 0.001 for each test versus control condition (pink, CM + no treatment; blue, CM + 10 mM 3-OBA; black, IncM + no treatment).

We next tested the influence of 10.0 mM 3-OBA treatment in lactate production upon different microenvironmental conditions. Thus, we considered: (1) the basic composition of the cell culture media (complete—containing glucose, glutamine and pyruvate—and incomplete—free of glucose, glutamine and pyruvate), and (2) the addition of 10 mM lactate to the cell culture media (lactate containing and non-containing media). The results are depicted in Figure 6. No apparent differences in extracellular lactate levels were observed when HT1376 cells were deprived of essential nutrients, but T24 cells produced significantly more lactate, regardless of 3-OBA treatment. As expected, extracellular lactate levels were significantly increased when 10 mM lactate was added to the cell culture media, independently of the presence of 3-OBA. In HT1376 cells, 3-OBA treatment decreased lactate production, both when the cells were cultured in complete or incomplete medium (differences were significant regarding the incomplete medium), which was not noted in T24 cell line. Presence of lactate in the culture medium neutralized those differences.

## 3. Discussion

3-Hydroxybutyrate is a ketone body produced endogenously under low-carbohydrate conditions and serves as an alternative energy source for various tissues. Beyond its metabolic role, 3-OBA influences signaling pathways related to cell growth and oxidative stress and has attracted attention for its therapeutic potential in neurological disorders, metabolic diseases, and cancer [32]. It has also been proposed as an antagonist of GPR81 [23], a lactate receptor upregulated in several tumors [28]. However, the precise role of 3-OBA in cancer and its validity as GPR81 antagonist remain unclear.

This study investigated the functional effects of 3-OBA in urothelial bladder cancer. Two muscle-invasive UBC cell lines demonstrated consistent responses to 3-OBA treatment, exhibiting reproducible anticancer effects across different experimental assays. A significant decrease in cell viability in a dose-dependent manner was observed, as well as decreased cells’ proliferation, invasion and migration, and a tendency for UCB cells’ accumulation in G0/G1 and G2/M phases upon 5 and 10 mM dosages. 3-OBA significantly hampered the growth and proliferative capabilities of UBC microtumors, concomitant with diminished neovascularization. Thus, in this study 3-OBA seemed to inhibit tumor progression through multiple cellular mechanisms related to proliferation, cell cycle dysregulation, angiogenesis, and dissemination. It is important to note that although the results of the in vitro assays seem to indicate that 3-OBA effects are dose-dependent, they are not time-dependent, because viability and migration assays did not reveal any difference among 24 h and 48 h of exposure of the cell lines to 3-OBA. This suggests that the inhibitory impact of 3-OBA on these UBC cells’ behaviors may occur early and does not increase with prolonged exposure within this timeframe, possibly indicating a plateau effect where the maximum achievable inhibition by 3-OBA on cell viability and migration is reached quickly. A total of 48 h of exposure did not add substantial additional cytotoxic or antimigratory effects already seen at 24 h, probably due to cellular adaptation or saturation of signaling pathway inhibition. This observation is clinically relevant, as it suggests shorter treatment durations may suffice for therapeutic effects targeting GPR81-related pathways in muscle-invasive bladder cancer.

The effects of 3-OBA in bladder cancer had not been previously described, to our knowledge, making our study the first to characterize its functional impact in UBC models. While direct studies of 3-OBA’s anticancer action in UBC are lacking, indirect evidence identified OXCT1, the mitochondrial enzyme responsible for 3-OBA catabolism, as significantly elevated in gemcitabine-resistant UBC. This enzyme drove increased mitochondrial respiration, ATP production and nucleotide synthesis in chemoresistant UBC cells, and its knockout restored chemosensitivity [33]. In line with these results, in pancreatic ductal adenocarcinoma high OXCT1 expression correlated with short relapse-free survival upon gemcitabine treatment; its overexpression decreased apoptosis upon gemcitabine exposure, while OXCT1 knockdown reversed resistance [34]. Thus, both studies highlight a possible metabolic role of OXCT1 in supporting an aggressive, treatment-resistant phenotype. Since 3-OBA is a natural substrate of OXCT1, its availability likely fuels OXCT1-mediated ketone body metabolism, which enhances energetic and biosynthetic capacity in cancer cells, thus promoting tumor progression, moreover, elevated 3-OBA levels could potentiate OXCT1-driven metabolic pathways linked to oncogenic signaling, as OXCT1 activity maintains ketone body homeostasis and activates pro-survival pathways such as NF-κB signaling via complex molecular crosstalk [35]. Thus, these data seem to indicate that 3-OBA indirectly favors cancer growth, which is in contrast to what we have observed in the present study. Additionally, Badameh et al. have recently shown that under low-glucose hypoxia, 3-OBA directly increased proliferation of colorectal cancer (CRC) cells in vitro [36]. However, the compound did not consistently impact proliferation or response to chemotherapy or radiation of breast cancer cells, particularly in normoglycemic conditions [21].

The above-mentioned studies align with the tumor-promoting effects of 3-OBA, but there are also multiple reports of its tumor-suppressive activity, similarly to the results presently obtained by us. 3-OBA inhibited viability, tumor growth, metastasis, epithelial-to-mesenchymal transition and stemness while inducing apoptosis in non–small cell lung cancer (NSCLC) [37], prostate cancer [38] and glioma [39]. The proliferation of colonic crypt cells in CRC mouse models was reduced upon 3-OBA administration, either via diet or as a direct supplement, mimicking effects of ketogenic diets. Single-cell RNA sequencing data of tissue sections from CRC patients demonstrated the same tendency of decreased cancer cells’ proliferation in patients with higher serum 3-OBA levels [40,41]. Reports also exist on the association of 3-OBA with a favorable outcome to chemotherapy, both in vitro and in vivo [42,43,44]. It resensitized oxaliplatin-resistant CRC cells; clinical patient data showed that lower serum 3-OBA levels correlated with resistance to oxaliplatin [43]. In hepatocellular carcinoma, the compound enhanced cisplatin-induced apoptosis through a putative epigenetic chemosensitizing mechanism [42]. Moreover, combined with agents like metformin or PD-1 inhibitors, 3-OBA enhanced CD8^+^ T cell activity in preclinical models. Through activation of HCAR2 (hydroxycarboxylic acid receptor 2), it suppressed NF-κB signaling, reducing inflammation and enhancing anti-tumor immune responses [23,45,46].

Thus, while 3-OBA anticancer properties have been evidenced by epigenetic regulation (via HDAC inhibition), oxidative stress and energy restriction mimicry, chemotherapy sensitization, and immunomodulation, the fact that it may serve as an alternative fuel for tumors under hypoxia and/or low glucose conditions, that may even induce stromal ketogenesis [47], highlights the opposite. Outcomes seem to vary with tumor type, microenvironment and therapeutic context, as well as being dose-dependent. 3-OBA may be cytotoxic at high doses, but physiological concentrations (~1–3 mM) may support cancer cell survival, particularly under hypoxic or nutrient-restricted conditions. The dual nature of 3-OBA underscores the importance of tumor stratification in metabolic therapies.

In the present study we used 5 and 10 mM 3-OBA doses in the functional assays and in the CAM model, as these were the lowest doses to promote a significant decrease in cells’ viability. These are commonly used in cell culture experiments to model ketosis-like or high ketone environments [48,49]. At these concentrations 3-OBA levels are higher than physiological fasting levels, but still within pathophysiological ranges (e.g., diabetic ketoacidosis) [50,51]. On the other hand, in vitro dosing does not fully mimic in vivo pharmacokinetics/dynamics; high doses might be needed in vitro to observe effects due to media dilution and the absence of intricate metabolic interactions. In fact, while the in vitro assays showed concentration-dependent effects of 3-OBA due to direct, controlled exposure of UBC cells to the compound, concentration-dependence was lost in the CAM assay, as the results do not show any difference between the 3-OBA concentrations that were used (5 and 10 mM). The superior biological complexity of the CAM model over the in vitro model probably blunted the dose-dependent responses that were seen under controlled in vitro conditions. Host factors of the CAM, including a rich vascular network, that further elaborate the extracellular microenvironment, coupled to pharmacokinetics and local compound distribution, as well as possibility of saturation of the target receptor or pathways, should be considered when comparing the results from both models. Understanding these differences is crucial for translating preclinical findings into optimal therapeutic dosing in bladder cancer. Still, more complex preclinical models and data at the UBC patient level are necessary to validate our results. Nevertheless, we used two muscle-invasive UBC cell lines that exhibit distinct genetic and molecular backgrounds, as well as differences in activated signaling pathways and metabolic activities. T24 is aligned with the FGFR3/CCND1 molecular subtype of muscle-invasive bladder carcinoma (HRAS-driven), Hedgehog-dependent (relies on the presence and activity of Hedgehog ligands to promote cancer growth, stemness, and drug resistance), glycolytic but metabolically less extreme than HT1376 cell line, which harbors genetic alterations that suggest a more invasive and metastatic phenotype (TP53/RB1/PTEN loss), is highly glycolytic and Hedgehog-independent [52,53,54]. Their cisplatin-resistant (CR) sublines underscore metabolic shifts that could impact drug sensitivity; thus, it would be interesting to further explore 3-OBA effects in these CR sublines. HT1376-CR exhibits a stronger metabolic rewiring toward glycolysis and lipogenesis than T24-CR, suggesting greater metabolic plasticity and stress adaptation [54]; this fact, coupled to the intrinsic invasive phenotype of HT1375 cells, may explain the absence of significant differences in the 3-OBA invasion assay of this cell line, in opposition to T24, that showed a dose-dependent decrease in invasive cells. T24 may represent a glycolytic-dominant model less prone to utilize ketones, while HT1376 may adapt metabolically to ketone supplementation or even deprivation.

The investigation of the putative link between the effect of 3-OBA on GPR81 signaling was the second aim of this work. 3-OBA has been widely used experimentally as a GPR81 antagonist in cancer [23] and ischemia [55] studies, often showing functional effects opposite to those of lactate or GPR81 agonists. Pharmacologically, 3-OBA is a known ligand of HCAR2 rather than HCAR1 (GPR81), raising concerns that its cellular effects may not reflect GPR81 blockade [31]. As previously mentioned, GPR81 is a G protein–coupled receptor activated by extracellular lactate. It is overexpressed in many cancers—including breast, pancreatic, lung and liver tumors—where it promotes tumor growth, survival, angiogenesis, and immune evasion by responding to high lactate levels in the tumor microenvironment. Its expression is linked to metabolic reprogramming and chemoresistance [27,28]. Prognostically, GPR81 can be associated with either better or worse outcomes depending on cancer type [56,57,58]. To our knowledge, there is no evidence of the prognostic role of GPR81 in bladder cancer, thus we performed a clinicopathological and survival analysis on a cohort of 53 UBC patients as a supplementary objective. The data showed a favorable clinicopathological profile for GPR81 overexpressing cases, although the differences did not reach statistical significance in the survival plots (data in Supplementary Materials).

Regarding the treatment of the UBC cell lines with 3-OBA, the control and 3-OBA treated groups did not show any significant GPR81 expression differences. In accordance, GPR81-related biomarkers like ERK, phospho-ERK, LDHA, MCT1/4 and CD147 were similarly expressed among the different groups, which indirectly denotes absence of any antagonistic activity. Evidence indicates that upon activation by agonist binding, GPR81 couples to the Gi protein (a subtype of heterotrimeric guanine nucleotide-binding proteins involved in cell signal transduction) and initiates the Gi signaling pathway: the Giα subunit of the Gi protein releases GDP and binds GTP, switching to an active state and thus activating downstream effectors such as ERK1/2 [59]; lactate-induced stimulation of GPR81 promoted activating phosphorylation of ERK1/2 in several studies, leading to downstream signaling [60,61,62]. In glycolytic cancer cells, LDHA produces lactate from pyruvate and MCTs, coupled to their chaperone CD147, export lactate produced by glycolysis, which then acts in an autocrine/paracrine fashion to activate GPR81. In turn, GPR81 signaling boosts expression of MCT1 and MCT4, reinforcing lactate metabolism and signaling, and tumor aggressive traits by creating a positive feedback loop [27]. In a study using different cancer models in vitro, activation of GPR81 by extracellular lactate promoted expression of MCT1, MCT4 and CD147, while its knockdown decreased their expression [63]. Thus, our results point to the absence of any influence of 3-OBA in the GPR81 axis. Despite the fact that the present work seems to rule out GPR81 signaling from 3-OBA mechanism of action, deeper mechanistic studies are necessary to confirm our results and to investigate 3-OBA effects in alternative signaling pathways.

We also challenged the UBC cell lines with different environmental conditions upon 10 mM 3-OBA treatment to check whether this would influence extracellular lactate levels; nutrient-rich versus nutrient-deprived media were used, as well as presence or absence of exogenous lactate. As expected, extracellular lactate levels increased significantly upon addition of 10 mM lactate, independent of 3-OBA treatment. Interestingly, in the absence of exogenous lactate, HT1376 cells did not show significant changes in lactate production between nutrient conditions, whereas T24 cells exhibited a marked increase in lactate release under nutrient deprivation—suggesting a rapid upregulation of glycolysis under nutrient stress in T24 cells, and a possible utilization of alternative metabolic pathways (e.g., fatty acid oxidation or amino acid catabolism) that do not significantly alter lactate output by HT1376 cells. Notably, 3-OBA significantly reduced lactate levels in HT1376 cells under nutrient deprivation, while no such effect was observed in T24 cells. 3-OBA is a monocarboxylate and is known to enter cells via MCTs, particularly MCT1. Thus, it might be competing with lactate for MCT transport, reducing lactate export, altering intracellular redox balance (via effects on NAD+/NADH), indirectly impairing LDHA activity, and impacting mitochondrial metabolism, leading to suppression of glycolysis. This suggests that differences in 3-OBA uptake between HT1376 and T24 cells could contribute to the observed variation in lactate response. HT1376 cells may express or utilize MCTs that favor 3-OBA uptake more effectively, increasing its intracellular concentration and metabolic impact. In contrast, T24 cells—potentially with higher MCT4 expression, as seen in the immunofluorescence images, which favors lactate efflux—may accumulate less 3-OBA, explaining their relative insensitivity. The addition of lactate to the medium neutralized these differences in both cell lines. Given that Western blot and immunofluorescence images show that HT1376 cells express lower levels of GPR81 compared to T24, it is unlikely that 3-OBA acts via specific GPR81 antagonism, and their effects are probably context-dependent. Thus, these results underscore the cell line-specific nature of lactate regulation and suggest that metabolic context, MCTs expression, and possibly alternative pathways all contribute to the observed response to 3-OBA.

Collectively, the results of the present study emphasize the potential of the anticancer properties of 3-OBA as a metabolic modulator in bladder cancer. By targeting lactate handling and altering tumor cell metabolism, 3-OBA may serve as a valuable therapeutic adjunct—particularly in metabolically less flexible tumors. Further studies are warranted to clarify its mechanism of action and to define patient subgroups most likely to benefit from 3-OBA–based interventions.

## 4. Materials and Methods

### 4.1. Cell Lines and Cell Culture Conditions

Two muscle invasive UBC cell lines, T24 and HT1376, were used in the experiments. These cell lines were acquired from the American Type Culture Collection and authenticated by short tandem repeat analysis. The cells were cultured as monolayers in Iscove’s Modified Dulbecco’s Medium (IMDM, PAN-Biotech^TM^, Aidenbach, Germany) supplemented with 1% antibiotics (penicillin/streptomycin solution, GRiSP, Porto, Portugal) and 10% fetal bovine serum (FBS, PAN-Biotech^TM^, Aidenbach, Germany), unless stated otherwise, and maintained at 37 °C in a humidified atmosphere with 5% CO_2_. The optimal seeding cell density was determined before undergoing each experiment. 3-OBA (Sigma-Aldrich^®^, St. Louis, MO, USA) or vehicle control (0.9% saline) diluted in IMDM were used in all of the assays.

### 4.2. Cell Viability Assay

The Sulforhodamine B (SrB, TOX-6, Sigma-Aldrich^®^, St. Louis, MO, USA) assay was used to assess the UBC cells’ response to increasing concentrations of 3-OBA (2.5, 5, 10, 20, 40 and 80 mM) after 24 and 48 h of treatment, as well as the impact of various microenvironmental conditions maintained for 48 h upon treatment with 10 mM 3-OBA. Cells were plated in triplicate in 48-well plates (T24—1.5 × 10^4^ cells/well, HT1376—2.55 × 10^4^ cells/well) using complete IMDM. After overnight (ON) adherence, the medium was replaced with (a) increasing concentrations of 3-OBA in FBS-free IMDM; (b) complete IMDM or Dulbecco’s Modified Eagle Medium without glucose, glutamine, and pyruvate (DMEM, Gibco, Thermo Fisher Scientific, Waltham, MA, USA), both with or without 10 mM lactate, and with or without 10 mM 3-OBA. After the respective treatment periods, cells were incubated with 10% trichloroacetic acid at 4 °C for 1 h, and the SrB assay was then performed, according to the instructions from the manufacturer. A Varioskan^®^ Flash (Thermo Fisher Scientific, Waltham, MA, USA) spectrophotometer was used to measure absorbance (490 nm). The resulting data were expressed as the percentage of cell viability normalized to the control condition, considering the results of at least three independent experiments.

### 4.3. Cell Proliferation Assay

The 5-bromo-2′-deoxyuridine (BrdU) immunoassay using the Cell Proliferation ELISA, BrdU, colorimetric kit (Roche Applied Sciences, Penzberg, Germany) was performed to quantify UBC cells’ proliferation upon 3-OBA treatment. After seeding T24 (1 × 10^4^ cells/well) and HT1376 (1.5 × 10^4^ cells/well) cells in 96-well plates in complete medium and allowing them to adhere ON, the medium was replaced by 5 and 10 mM 3-OBA in FBS-free IMDM for 48 h. The cell proliferation assay was performed according to the manufacturer’s instructions, with the following specific conditions: a concentration of 10 μM BrdU was used to stain the cells for 8 h, cells were incubated with the antibody anti-BrdU-POD (1:100 dilution) for 90 min at room temperature (RT), and with 100 µL/well of substrate solution for a maximum of 30 min or until color development. Absorbance readings from quadruplicates of at least three independent experiments were obtained in the Varioskan^®^ Flash spectrophotometer (Thermo Fisher Scientific, Waltham, MA, USA) at 450 nm. The resulting data were expressed as percentage of cell proliferation normalized to the control condition.

### 4.4. Cell Migration Assay

The wound healing assay was performed to assess the ability of the UBC cells to migrate upon 3-OBA treatment. T24 and HT1376 cells were plated in 6-well culture and incubated in complete IMDM until reaching 90% confluency. A sterile pipette tip was used to make two wounds per well. Cells that became detached were removed with a gentle PBS 1× wash, and 5 or 10 mM 3-OBA in FBS-free IMDM was added to the wells. Photographs were taken at this timepoint (0 h) and at 24 and 48 h of incubation to 4 reference wound sites under a phase contrast microscope (Olympus^®^ IX51, Tokyo, Japan), being analyzed with the beWound v1.7 software. The resulting data were expressed as percentage of cell migration normalized to the control condition.

### 4.5. Cell Invasion Assay

The invasion ability of the UBC cells upon treatment with 3-OBA was assessed using Corning^®^ BioCoat™ Matrigel^®^ Invasion Chambers (Corning^®^, New York, NY, USA), according to the manufacturer’s instructions. The bottom of the lower chambers was filled with a chemoattractant (IMDM with 10% FBS), and the inserts containing T24 or HT1376 cells (2 × 10^4^ cells/insert) in FBS-free IMDM with either 5 or 10 mM 3-OBA were placed inside the wells. Non-invading cells were removed after 24 h of treatment incubation, and invading cells were fixed (100% methanol) to the inserts’ membrane and stained (Giemsa dye). A stereomicroscope (Olympus^®^ S2 × 16, Tokyo, Japan) was used to obtain images of the membranes. The ImageJ software (version 1.50i) was used to count the cells from at least three independent assays. The resulting data were expressed as percentage of invading cells normalized to the control condition.

### 4.6. Cell Cycle and Cell Death Analysis

Propidium iodide (PI) or PI and Annexin V stainings were performed to analyze the UBC cells cycle distribution and cell death ocurrence (respectively) by flow cytometry upon 3-OBA treatment. After seeding T24 (1 × 10^5^ cells/mL) or HT1376 (1.6 × 10^5^ cells/mL) cells in T25 culture flasks in complete medium and allowing them to adhere ON, cells were treated with fresh complete medium containing 5 or 10 mM 3-OBA for 48 h. For cell cycle analysis, at least 2 × 10^6^ cells were collected, pelleted, washed with PBS 1×, and ressuspended in cold ethanol (70% *v*/*v*; 30 min, 4 °C). The following labeling solution was used: 20 µg/mL PI (Invitrogen^TM^, Waltham, MA, USA), 250 µg/mL RNase A (Invitrogen^TM^, Waltham, MA, USA) and 0.1% Triton X-100 diluted in PBS 1×. The samples were incubated in the dark at 50 °C for 1 h. For cell death analysis, floating and adherent cells were collected, peletted, washed with PBS 1×, and incubated in the dark at RT for 15 min in 5 μL FITC annexin V (BD Pharmingen™, BD Biosciences^®^, Franklin Lakes, NJ, USA) and/or 10 μL PI (Invitrogen^TM^, Waltham, MA, USA) diluted in binding buffer. Labeled cells were counted in a FACS LSRII flow cytometer (BD Biosciences^®^, Franklin Lakes, NJ, USA); at least 10,000 events were considered. The FlowJo software (version 10, Tree Star, Inc., San Francisco, CA, USA) was used to analyze the cell cycle distribution and cell death from at least three independent assays. The resulting data were expressed as percentage of cells distributed by the cell cycle phases (G0/G1, S, G2/M) or cell death events (necrotic, viable, early apoptotic, late apoptotic/necrotic cells) relative to the control condition.

### 4.7. Chick Chorioallantoic Membrane (CAM) Assay

The CAM assay was performed to analyze the influence of 3-OBA treatment on tumor growth and angiogenesis occurrence. The assay initiated by incubating fertilized chicken eggs at 37 °C and 80% humidity; those conditions were maintained from day (D) 0 to D17. Access to the embryo was obtained by performing a small opening in each egg and the eggs were sealed with duct tape before returning to the incubator. The following procedures were undertaken at specific days: (D3) the shells of the eggs were separated from the CAM and embryo viability was confirmed; (D9) T24 or HT1376 cells’ suspensions (2 × 10^6^ cells in 10 µL Matrigel) were grafted on the CAM; (D13) the tumors were photographed and treatment (5 or 10 mM 3-OBA in FBS-free IMDM) or control conditions were administered; (D17) the tumors were photographed, the embryos were sacrificed (CO_2_ anesthesia and thermal shock at −80 °C), the tumor-containing CAMs were dissected and photographed. All “in ovo” and “ex ovo” images were obtained under a stereomicroscope (Olympus^®^ S2 × 16,Tokyo, Japan). Tumor perimeter and blood vessels’ countings were obtained in the ImageJ software (version 1.50i). The resulting data were expressed as tumor growth (fold change) or number of blood vessels relative to the control condition. Moreover, UBC cells’ proliferation and tumor vascularization were assessed in 4 μm thick sections of the CAM excised tumors by immunohistochemistry, using antibodies for Ki-67 (1:500, ab16667, AbCam, Cambridge, UK) and lectin (1:100, B-1305-2, Vector Laboratories, Newark, CA, USA) detection, respectively, and the corresponding Thermo Scientific™ Lab Vision™ UltraVision™ ONE Detection System: HRP Polymer (Thermo Fisher Scientific, Waltham, MA, USA) and Large Volume Detection System: anti-Polyvalent, HRP (Thermo Fisher Scientific, Waltham, MA, USA) detection kits. A lymphoma and a CAM section with known positivity for Ki-67 and lectin staining (respectively) were used as positive controls, while negative controls were obtained by omitting the primary antibodies. The reactions were visualized using a Liquid 3,3′-Diaminobenzidine (DAB)-containing substrate chromogen system (Lab Vision™ DAB Plus Substrate Staining System, Thermo Fisher Scientific, Waltham, MA, USA). Immunoreactivity was semi-quantitatively evaluated in hotspot areas under an Olympus^®^ BX61 (Tokyo, Japan) microscope, using a grading system based on the amount of cells expressing either Ki-67 or lectin (0, 0% of positive cells; 1, <5% of positive cells; 2, 5–50% of positive cells; 3, >50% of positive cells).

### 4.8. Cell Lysis, Protein Extraction and Western Blotting

T24 and HT1376 cells were plated to achieve 70% confluency in the following day. They were then treated with 5 or 10 mM 3-OBA (or 0.9% saline) in FBS-free IMDM for 48 h. Cell lysis was performed by scraping and incubation in a lysis buffer (with protease inhibitors from Roche Applied Sciences, Penzberg, Germany) for 10 min on ice. Protein content was extracted by centrifugation at 13,000 rpm for 15 min at 4 °C, and protein quantification was performed using the colorimetric Bradford assay (Sigma-Aldrich^®^, St. Louis, MO, USA).

For the Western blot of the samples, 20 µg of total protein was loaded and separated on 10% polyacrylamide gels by SDS-PAGE in the Mini-PROTEAN^®^ Tetra System (Bio-Rad Laboratories, Hercules, CA, USA). Subsequently, the proteins were transferred to nitrocellulose membranes using the Trans-Blot^®^ Turbo™ Transfer System (Bio-Rad Laboratories, Hercules, CA, USA) for 30 min at 25 V. Following a brief incubation with Ponceau S solution, the protein-containing membranes were blocked in 5% milk in TBS + 0.05% Tween 20 (TBS-T) for 1 h at RT. The membranes were then incubated ON at 4 °C with specific primary antibodies (or loading controls, Table S2), with the appropriate secondary antibodies (Table S2) for 1 h at RT, and with an enhanced chemiluminescence substrate (WesternBright^®^ Sirius^®^, Advansta Inc., San Jose, CA, USA); these steps were interspersed with washes in TBS-T. The Azure Sapphire Biomolecular Imager (Azure Biosystems) was used to detect the proteins of interest. Images were analyzed in the ImageJ software (version 1.50i).

### 4.9. Immunofluorescence

T24 and HT1376 cells were seeded (5 × 10^4^/well) in round coverslips, in 12-well plates containing complete IMDM medium, and allowed to adhere ON. Spent medium was then replaced by 5 or 10 mM 3-OBA (or 0.9% saline) containing FBS-free IMDM. After 48 h of treatment, cells were washed with cold PBS 1×, fixed and permeabilizated with cold methanol at −20 °C for 20 min. After a blocking step (30 min at RT in 5% BSA), the cells were incubated ON (at 4 °C) with specific primary antibodies (Table S2), followed by incubation with fluorescence-conjugated secondary antibodies (Table S2) for 1 h at RT in the dark, and mounting in Fluoroshield^TM^ with DAPI (4′,6-diamidino-2-phenylindole, Sigma-Aldrich^®^, St. Louis, MO, USA). Images were vizualized in an Olympus^®^ BX61 fluorescence microscope (Tokyo, Japan).

### 4.10. Colorimetric Analysis of Extracellular Lactate

A commercial colorimetric kit (Spinreact, Girona, Spain) was used to analyze the levels of extracellular lactate after incubating the UBC cells in different microenvironmental conditions for 48 h. Thus, after seeding T24 (1.5 × 10^4^ cells/well) and HT1376 (2.55 × 10^4^ cells/well) cells in 48-well plates in complete medium and allowing them to adhere ON, the medium was replaced by complete IMDM or Dulbecco’s Modified Eagle Medium without glucose, glutamine, and pyruvate (DMEM, Gibco, Thermo Fisher Scientific, Waltham, MA, USA), both with or without 10 mM lactate, and with or without 10 mM 3-OBA. Lactate levels in the spent medium were analyzed according to the instructions from the manufacturer. Absorbance readings from triplicates of at least three independent experiments were obtained in the Varioskan^®^ Flash spectrophotometer (Thermo Fisher Scientific, Waltham, MA, USA) at 490 nm. The SrB assay was performed for biomass quantification. The resulting data were expressed as total μg/biomass.

### 4.11. Statistical Analysis

Raw results from the in vitro and the in vivo assays were stored in Excel files and the statistical analysis was performed in the GraphPad Prism 8.4.2. software, using unpaired two-tailed Student’s *t*-test, and one- or two-way ANOVA followed by Dunnet’s, Tukey’s or Bonferroni multiple comparison post hoc tests. Results are presented as normalized means ± standard error of the mean (SEM) of at least three independent assays (technical and biological replicates). *p* values < 0.05 were considered significant (* *p* < 0.05, ** *p* < 0.01, *** *p* < 0.005 and **** *p* < 0.001).

## Data Availability

All data generated during the study are presented within the article/Supplementary Material. Additional inquiries can be addressed to the corresponding author.

## Supplementary Materials

The following supporting information can be downloaded at: https://www.mdpi.com/article/10.3390/molecules30234624/s1, Figure S1: Quantification of the Western blot results from Figure 5. Figure S2: Representative bladder tissue sections from urothelial bladder carcinoma patients showing increasing intensities of GPR81 cytoplasmic immunoexpression in the malignant cells. Table S1: Association between the immunoexpression of GPR81 and the clinicopathological data of urothelial bladder carcinoma patients (n = 53). Table S2: Antibodies used for Western blotting (WB) and immunofluorescence (IFC). Refs. [64,65,66] are cited in the Supplementary Materials.
