# Supplementary material for: Functional and Mechanistic Insights of 3-Hydroxybutyrate (3-OBA) in Bladder Cancer
Source: Molecules. 2025 Dec 2;30(23):4624. doi: 10.3390/molecules30234624 (PMC12693350; doi:10.3390/molecules30234624)
Supplement: Supplementary file 1 [file molecules-30-04624-s001.zip › molecules-3833738-supplementary.pdf]

molecules

MDPI

Supplementary Material

Functional and Mechanistic Insights of 3-hydroxybutyrate (3-OBA) in bladder cancer

Ana Siva [1,2*], Ana Mafalda Félix [1,2*], Céline Saraiva Gonçalves [1,2], Adhemar Longatto-Filho[1,2,3,4], Fátima Baltazar [1,2], Julieta Afonso [1,2#]

[1] Life and Health Sciences Research Institute (ICVS), University of Minho, Campus of Gualtar, 4710-057 Braga, Portugal; id10583@alunos.uminho.pt (A.S.); mafaldafelix98@gmail.com (A.M.F.); celinegoncalves@med.uminho.pt (C.S.G.); longatto@med.uminho.pt (A.L.-F.); fbaltazar@med.uminho.pt (F.B.); julietaafonso@med.uminho.pt (J.A.)
[2] ICVS/3B's—PT Government Associate Laboratory, 4710-057 Braga, Portugal
[3] Laboratory of Medical Investigation (LIM14), Faculty of Medicine, São Paulo State University, São Paulo 01049-010, SP, Brazil
[4] Molecular Oncology Research Center, Barretos Cancer Hospital, Barretos 14784-400, SP, Brazil
* These authors contributed equally to the work
\# Correspondence: julietaafonso@med.uminho.pt

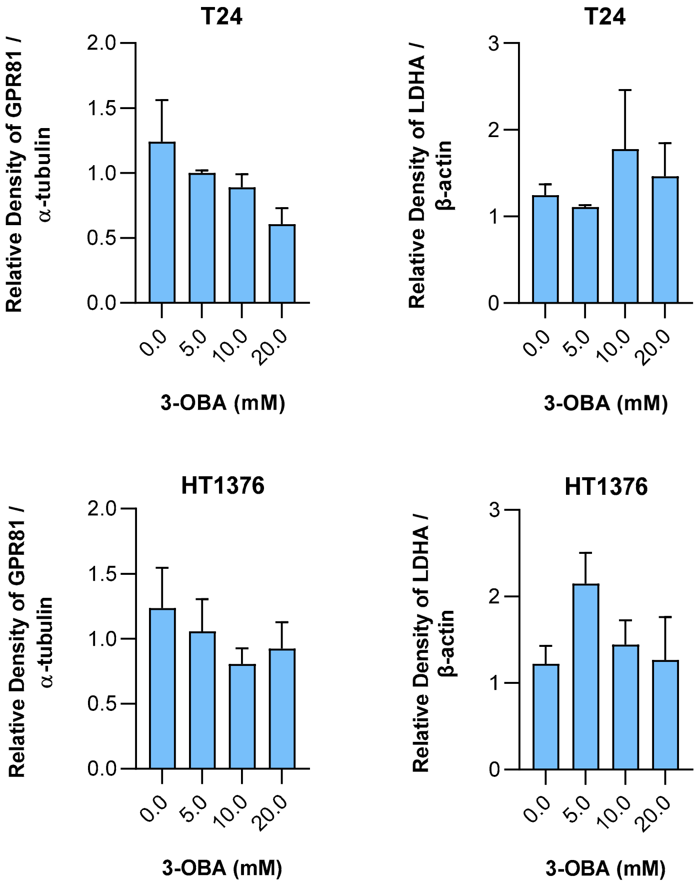

Figure S1: Quantification of the Western blot results from Figure 5. Statistical significance was estimated by one-way ANOVA followed by Dunnet's multiple comparison post hoc test. p>0.05 for each 3-OBA concentration versus control condition (0.0 mM 3-OBA).

Clinicopathological and prognostic significance of GPR81 expression in tissue samples from UBC patients

To the best of our knowledge, there are no studies investigating the clinicopathological and prognostic significance of GPR81 expression levels in UBC patients. Thus, we analyzed its immunoexpression in 53 sections from UBC patients and 7 non-UBC bladder controls. We started by confirming that patients with highly aggressive tumors had significantly worse DFS and OS rates (data not shown). When expressed, GPR81 was observed in the cytoplasm of both non-malignant and malignant cells (Figure S2). No significant differences were observed regarding GPR81 immunoexpression in non-neoplastic bladder tissues and UBC samples (data not shown). All the non-muscle invasive tumors (n=11) expressed GPR81, this difference being significant when compared with the muscle-invasive cases (Table S1). Although it seems that GPR81 is associated with a favorable clinicopathological profile, it did not influence DFS or OS rates. In the group of platinum-based treated UBC patients (n=17), GPR81 did not discriminate between a better or a worse prognosis (data not shown).

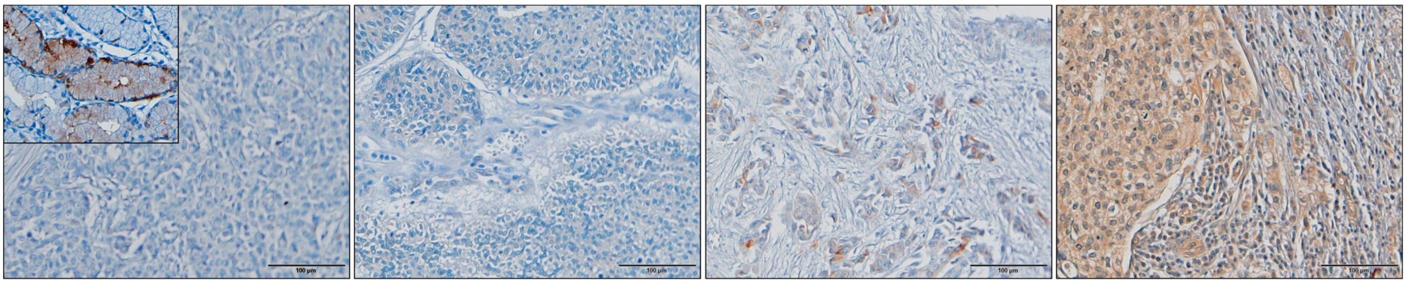

Figure S2. Representative bladder tissue sections from urothelial bladder carcinoma patients showing increasing intensities of GPR81 cytoplasmic immunoexpression in the malignant cells. In "Intensity 3" case, positive stromal fibers and blood vessels are present. Inset regarding the positive control (gastric carcinoma section). Original magnification 100x.

Table S1. Association between the immunoexpression of GPR81 and the clinicopathological data of urothelial bladder carcinoma patients (n=53).

	n	GPR81 positive (%)	*p*
**Age**			0.416
≤ 71 years	28	16 (57.1)	
> 71 years	25	17 (68.0)	
**Gender**			0.695
Male	45	27 (60.0)	
Female	8	6 (75.0)	
**TNM stage**			0.006
pTa, pT1, pTis	11	11 (100.0)	
pT2	11	7 (63.6)	
pT3, pT4	31	15 (48.4)	
**Grade and Type of Lesion**			0.006
NMIP UC, HG	9	9 (100.0)	
NMI UC in situ	2	2 (100.0)	
MI UC	42	22 (52.4)	
**LVI**			0.185
Negative	30	21 (70.0)	

Positive	23	12 (52.2)	
**Loco-regional metastases**			0.187
Negative	35	24 (68.6)	
Positive	18	9 (50.0)	

p values from Pearson Chi-square or Fisher's exact tests

Materials and Methods

a) Patients, Tissue samples and Immunohistochemistry

Fifty three patients diagnosed with UBC who were submitted to surgical resection at the Portuguese Institute of Oncology, Porto, from January 1996 to December 2005, were included in this study; the Ethics Committee of the institution granted previous approval to the study (approval code CES-IPOFG-EPE 86/2017), which was conducted strictly according to the guidelines of the Declaration of Helsinki. The medical records of the patients were consulted to obtain clinical and follow-up data, after which the respective formalin-fixed paraffin-embedded surgical specimens were collected. Patients with low-grade UBC, variant histology UBC, squamous cell or adenocarcinomas, and/or with a short follow-up period, and/or with samples innapropriate for histological evaluation were excluded from the study. The median age of the UBC patients was 71 years (range 44–82); regarding gender, 45 (75.0%) were male and 8 (13.3%) were female. Additionally, samples of the urinary bladder were obtained from 7 autopsy patients without known UBC history (non-UBC controls).

The guidelines of the College of American Pathologists were used to evaluate the surgical specimens [1]; the American Joint Committee on Cancer (AJCC) [2] and the World Health Organization (WHO) [3] classifications were used to categorize haematoxylin-eosin (H&E)-stained sections by standard histopathological examination (two independent pathologists), considering age, gender, TNM stage (three groups), grade and type of lesion (three groups), presence of lymphovascular invasion and that of locoregional metastasis (Table S1). All of the patients were submitted to radical cistectomy (RC), and 17 (32.1%) of them received cisplatin-based chemotherapy. The mean and the median follow-up periods were 45 and 24 months (range 1–154), respectively. Recurrence occurred in 40 (75.5%) patients, being defined as the reappearance of UBC (loco-regional or distant metastasis) more than 3 months after surgery. The time from surgery to recurrence or to death by UBC (or the last clinical assessment) defined disease-free (DFS) and overall survival (OS), respectively.

An immunohistochemistry protocol was used to stain GPR81 (SAB1300089 1:100; Sigma-Aldrich®, St. Louis, MO, USA) in representative 4μm-thick UBC and non-UBC sections. The Thermo Scientific™ Lab Vision™ UltraVision™ ONE Detection System: HRP Polymer (Thermo Fisher Scientific, Waltham, MA, USA) kit was used for detection. A gastric cancer section with known positivity for GPR81 was used as positive control, while negative controls were obtained by omission of the primary antibody. The reactions were visualized using a Liquid 3,3'-Diaminobenzidine (DAB)-containing substrate chromogen system (Lab Vision™ DAB Plus Substrate Staining System, Thermo Fisher Scientific, Waltham, MA, USA). Immunoreactivity was semi-quantitatively evaluated in hotspot areas for membrane and/or cytoplasmic staining of urothelial cells under an Olympus® BX61 (Tokyo, Japan) microscope, using a grading system based on the percentage of stained cells (0, 0% positive; 1, < 5% positive; 2, 5-50% positive; 3, >50% positive) and the intensity of staining (0, negative; 1, weak; 2, intermediate; 3, strong). The sum of percentage and intensity defined the final score (cutoff = 3: score <3 negative and score ≥3 positive). GPR81 immunoreactivity (clustered as negative or positive) was also assessed in the urothelial cells' surrounding stroma (mostly fibroblasts and collagen fibers) and in the vascular structures.

b) Statistical Analysis

Regarding the immunohistochemistry results, the analysis was conducted using SPSS software for Windows, version 25. The frequency of GPR81 immunoreactivity in non-UBC and UBC tissues, as well as correlations with the clinicopathological parameters, were analyzed with Pearson's chi-square (χ^2) and Fisher's exact (> 20% cells with

expected frequencies < 5) tests. The DFS and OS rates were analyzed by the Kaplan–Meier method (differences assessed by the Log-Rank or Breslow tests); parameters showing p values <0.05 were considered significant.

c) References

1. Amin, M. B.; Srigley, J. R.; Grignon, D. J.; Reuter, V. E.; Humphrey, P. A.; Cohen, M. B.; Hammond, M. E. H., *Urinary bladder cancer protocols and checklists*. College of American Pathologists: Northfield, 2005.
2. Edge, S. B.; Byrd, D. R.; Compton, C. C.; Fritz, A. G.; Greene, F. L.; Trotti, A., *AJCC Cancer Staging Manual*. Springer Verlag: New York, 2010.
3. Eble, J. N.; Sauter, G.; Epstein, J. I.; Sesterhenn, I. A., *Pathology and Genetics of Tumours of the Urinary System and Male Genital Organs*. IARC Press: Lyon, 2004.

Table S2. Antibodies used for Western blotting (WB) and immunofluorescence (IFC).

	Antibody	Reference (Company)	WB dilution	IFC dilution
**Primary antibodies**	**CD147**	sc-71038 (Santa Cruz Biotechnology®)	-	1:500
	**ERK1/ERK2**	#4695 (Cell Signaling TECHNOLOGY®)	-	1:800
	**p-ERK1/ERK2**	#4370 (Cell Signaling TECHNOLOGY®)		1:400
	**GPR81**	sc-32647 (Santa Cruz Biotechnology®)	1:500	1:250
	**LDHA**	sc-100775 (Santa Cruz Biotechnology®)	1:1000	-
	**MCT1**	AB3538P (Chemicon®)	-	1:200
	**MCT4**	sc-50329 (Santa Cruz Biotechnology®)	-	1:500
**Secondary antibodies**	**m-IgGκ BP-HRP**	sc-516102 (Santa Cruz Biotechnology®)	1:2500	
	**IgG-HRP**	sc-2357 (Santa Cruz Biotechnology®)	1:2500	
	**Alexa Fluor® 594**	A11032 (Invitrogen™)		1:500
	**Alexa Fluor® 488**	A11008 (Invitrogen™)		1:500
**Loading controls**	**β-Actin**	sc-8432 (Santa Cruz Biotechnology®)	1:500	
	**α-Tubulin**	ab15246 (AbCam)	1:2500	
